# O.3.3-5 Is parkrun an equitable health intervention? A qualitative study exploring how a free, weekly, community-based physical activity initiative engages with underrepresented groups in the UK

**DOI:** 10.1093/eurpub/ckad133.160

**Published:** 2023-09-11

**Authors:** Helen Quirk

**Affiliations:** University of Sheffield, UK; h.quirk@sheffield.ac.uk

## Abstract

**Purpose:**

Underrepresented groups face unique and complex barriers to community-based physical activities. This study used the worldwide initiative *parkrun* to explore how it has engaged with underrepresented groups in the UK. It sought to explore the challenges faced by the organisation and its volunteers when striving for equity and outline the inclusive promotion strategies that could be used by *parkrun* and other community initiatives.

**Methods:**

Adult *parkrun* Ambassadors who fulfilled volunteer roles that involved ‘outreach’ to underrepresented groups across the UK were invited. Ten volunteers provided informed consent to be interviewed via telephone in April-July 2021. Interview transcripts were analysed using a thematic approach by the same researcher who conducted the interviews. Ethical approval was received from the University of Sheffield REC.

**Results:**

The Ambassadors had volunteering experience from two to five or more years. The outreach approaches implemented varied from opportunistic promotion within communities to strategic negotiations at higher decision-making levels. Ambassadors described a community-centred focus that ensured that existing community networks and assets were utilised when promoting *parkrun*. It was advantageous to identify influential ‘community connectors’ within the target community with power to mobilise others. These could be local people or organisations in more traditional positions of influence e.g., General Practices and schools. The volunteers conveyed many challenges to their attempts to increase engagement in *parkrun* by underrepresented groups. Limited personal and organisational capacity meant that the reach and impact of engagement was restricted. The Ambassadors would benefit from robust mechanisms for reflecting, evaluating, learning and monitoring progress to better understand what is working (or not) and why.

**Conclusion:**

*parkrun* takes a community-centred approach to outreach. Community initiatives like *parkrun* may benefit from a whole-system approach that is adaptive to local contexts, considers where the greatest opportunities for change are and fosters a learning mindset among those delivering the initiative. The findings only represent the views of ten Ambassadors interviewed and so further collaborative work with people from the underrepresented groups is needed to develop co-produced solutions to equitable participation.

**Funding:**

Funded by a National Institute for Health Research School for Public Health Research post-doctoral launching fellowship.

